# Osteopontin (OPN) Is an Important Protein to Mediate Improvements in the Biocompatibility of C Ion-Implanted Silicone Rubber

**DOI:** 10.1371/journal.pone.0098320

**Published:** 2014-06-09

**Authors:** Shao-liang Wang, Xiao-hua Shi, Zhi Yang, Yi-ming Zhang, Li-ru Shen, Ze-yuan Lei, Zhi-qing Zhang, Cong Cao, Dong-li Fan

**Affiliations:** 1 Department of Plastic and Cosmetic Surgery, Xinqiao Hospital, Third Military Medical University, Chongqing, People's Republic of China; 2 Southwestern Institute of Physics, Chengdu, Sichuan, People's Republic of China; 3 Institute of Neuroscience, Soochow University, Suzhou, Jiangsu, People's Republic of China; Bascom Palmer Eye Institute, University of Miami School of Medicine, United States of America

## Abstract

Medical device implants are drawing increasing amounts of interest from modern medical practitioners. However, this attention is not evenly spread across all such devices; most of these implantable devices can cause adverse reactions such as inflammation, fibrosis, thrombosis, and infection. In this work, the biocompatibility of silicone rubber (SR) was improved through carbon (C) ion implantation. Scanning electron microscopy (SEM), atomic force microscopy (AFM), X-ray photoelectron spectroscopy (XPS), and X-ray diffraction (XRD) results confirmed that these newly generated carbon-implanted silicone rubbers (C-SRs) had large, irregular peaks and deep valleys on their surfaces. The water contact angle of the SR surface decreased significantly after C ion implantation. C ion implantation also changed the surface charge distribution, silicone oxygen rate, and chemical-element distribution of SR to favor cell attachment. The dermal fibroblasts cultured on the surface C-SR grew faster and showed more typical fibroblastic shapes. The expression levels of major adhesion proteins, including talin-1, zyxin, and vinculin, were significantly higher in dermal fibroblasts cultured on C-SR coated plates than in dermal fibroblasts cultured on SR. Those same dermal fibroblasts on C-SRs showed more pronounced adhesion and migration abilities. Osteopontin (OPN), a critical extracellular matrix (ECM) protein, was up-regulated and secreted from dermal fibroblasts cultured on C-SR. Matrix metalloproteinase-9 (MMP-9) activity was also increased. These cells were highly mobile and were able to adhere to surfaces, but these abilities were inhibited by the monoclonal antibody against OPN, or by shRNA-mediated MMP-9 knockdown. Together, these results suggest that C ion implantation significantly improves SR biocompatibility, and that OPN is important to promote cell adhesion to the C-SR surface.

## Introduction

Bio-implant materials are widely used in reconstructing and repairing human organs damaged by injuries or degradation [1]. Silicone rubber (SR)-based materials have been used for many years, as they have excellent physiology inertia, high adsorption properties, high corrosion resistance, good chemical stability and high mechanical strength [2], [3]. It is also workable and convenient. However, there are still many tribulations when using this material. The intrinsically hydrophobic nature of SR surface makes cell adhesion almost impossible, causing problems like fibrous capsules, contracture formation, and displacement during long-term usage [4]–[6]. Poor cell adhesion on its surface allows a gap to form between the SR implant and surrounding tissues, this can lead to bacterial invasion [7].

The surface modification to improve the biocompatibility of SR is a common way of addressing this issue. In recent years, there have been many attempts [8]–[10]. For example, osteoblast cells attached more strongly and grew faster on silicone rubber coated with carbon nanotubes (CNTs) than on uncoated rubber [11]. Among the various techniques, ion implantation is versatile and attractive because several properties such as cyto-compatibility and corrosion resistance will be enhanced, whereas the favorable attributes including biomechanical properties can usually be preserved [12]–[15]. Several groups have reported the use of nitrogen and carbon plasma immersion ion implantation (N-PIII and C-PIII) to modify Ti-6Al-4V [16]. Ion implantation has been also applied on biomedical polymer materials, including PMMA, polytetrafluorethylene (PTFE) and others [17]. However, few papers discussing ion implantation on silicone rubber have been found, not to mention the mechanism of its possible cyto-compatibility improvement.

One possible explanation for the improvements in cell attachment and retention involves protein adsorption, which is known to determine the accessibility of implanted materials to cells [17]–[19]. Depending on the physical and chemical properties of their surfaces, the implant materials absorb ECM proteins, which work as ligands to bind to integrins and other receptors on the cell surface. The ligand-receptor interaction then forms a focal adhesion complex, which activates signal transduction through focal adhesion kinase. These signals provoke recombination of the cytoskeleton, involving adhesion-associated proteins including talin-1, vinculin, zyxin and others, which facilitates cell adhesion to the implanted materials, where these cells will migrate and proliferate [17]–[19].

In the current study, we successfully modified the surface of SR by carbon (C) ion implantation. We examined the effectiveness of C ion implantation by evaluating the results of surface characteristics and the cyto-compatibility. Results indicated that when human dermal fibroblasts were cultured on carbon-implanted silicone rubber (C-SR), they tended to grow faster and showed more typical fibroblast shapes than cells cultured under SR, they also demonstrated more pronounced adhesion and migration abilities. Meanwhile, adhesion-associated proteins including talin-1, vinculin and zyxin were up-regulated in these cells. These phenomena were found to be positively related with ion doses. In particular, we found that OPN, an ECM component and a soluble cytokine, worked as an important protein to mediate biocompatibility improvement of C ion-implanted silicone rubber. Possible mechanisms by which OPN might improve cyto-compatibility were also investigated. We found that MMP-9 is the downstream signal molecular of OPN, playing a role in cells migration. These results suggested that C ion implantation can improve the biocompatibility of SR considerably, and that OPN works as an important protein to promote cell adhesion on the surface of C-SR.

## Materials and Methods

### Silicone rubber (SR) preparation

Equal amounts (10 ml) of clinical-quality liquid SR-A and SR-B (Polydimethylsiloxane, GN501, viscosity 1500–2000, Chenguang Research Institute of Chemical Engineering, China) were mixed, stirred and slowly injected into a metal plate mold (100 mm×100 mm×0.5 mm). The mixture was then vacuumed under −0.1 MPa for 30 minutes. It was then allowed to solidify at room temperature for 5.5 h. Only pure silicone rubber sheets with smooth surfaces were used for further experiments. All operations were carried out under sterile conditions.

### SR surface modification

The surface of SR was modified by carbon ion implantation. Carbon atom is a compositional element of SR and is harmless to the human body. Carbon ions were implanted onto the surface of SR in a neutral and ionized alkaline bombardment-type heavy ion source, in which the ions were produced from a sintered carbon (99.99%). The silicone rubber surface was implanted with carbon ions at an ion energy of 10 keV and carbon ion doses of 1×10^15^ ions/cm^2^ (**C-SR-1**), 3×10^15^ ions/cm^2^ (**C-SR-2**) and 1×10^16^ ions/cm^2^ (**C-SR-3**). The current density during implantation less than 600 nA/cm^2^ and residual gas pressure was less than 1×10^−3^ Pa. All operations were carried out under sterile conditions.

### SR and C-SR surface observation and analysis

**Figure 1:**
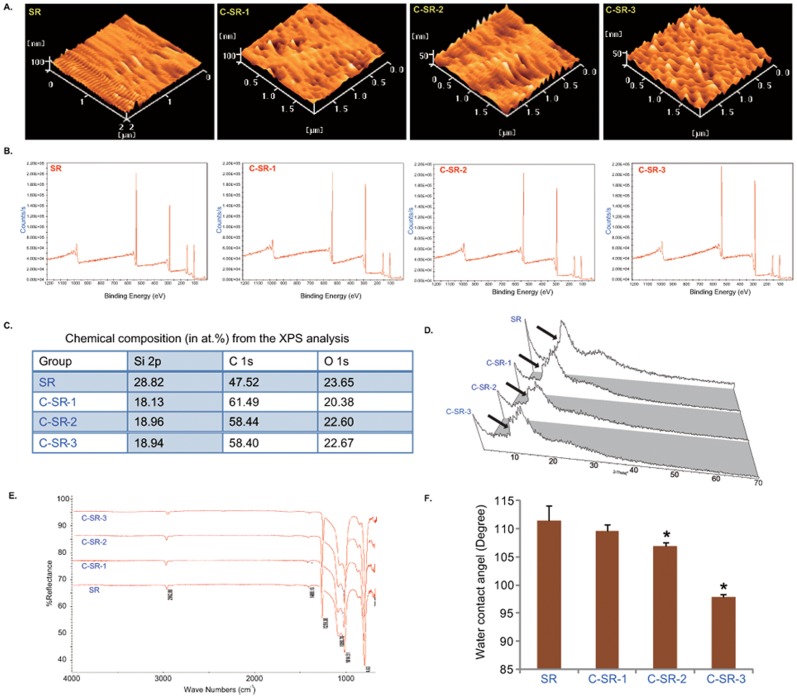
C ion implantation changes physical and chemical properties of SR surface. (A) AFM images show the micro-morphology of the surfaces of SR and three different C-SRs (C-SR-1, C-SR-2 and C-SR-3). C ion implantation changed the surface micro-morphology of SR. Note that with the ion implantation dose increased, the surface tended to be more uneven, but arranged regularly. (B and C) XPS analysis of the chemical composition of SR and three different C-SRs. C ion implantation changed the chemical composition of SR. (D) XRD analysis of SR and three different C-SRs. Results show that the XRD pattern of SR was changed after C ion implantation, indicating that new crystal structures may form. The difference is very small. (E) FTIR analysis of the formation of new bonding motifs. The spectrums for C-SRs were similar to that for pristine SR. (F) The water contact angle of SR and three different C-SRs. Note that C ion implantation significantly decreased water contact angle. *p<0.05 The difference was statistically significant when C-SR-2 or C-SR-3 was compared to SR. Experiments in this figure were repeated three times, similar results were obtained each time.

For scanning electron microscope (SEM, AMRAY 1000-B, Amray Inc, Bedford, Mass, USA) observations, SR and C-SRs were cut into 10 mm×10 mm squares and dried in 37°C. Afterwards, these squares were put into a vacuum pump to spray painting gold coat on the surface. SEM microscope was then applied to observe the surface micro-morphology and the fibroblast shape. The latter was also examined by atomic force microscopy (AFM) (SAP 400, Seiko Instrument Inc.). For the fourier transform infrared spectroscopy (FTIR) analysis, the SR and C-SRs were cleaned by dehydrated alcohol. And then the materials surface ingredients were examined by FTIR microscopy (Nicolet 470 spectrometer, Thermo Scientific), with the wave number at 4 cm^−1^, and the scan extent at 4000∼400 cm^−1^. For the X-ray Diffraction (XRD) detection, the SR and C-SRs squares were cleaned by dehydrated alcohol, non-destructive XRD (D/MAX 1200, Rigaku Inc.) was applied to reveal information about crystallographic compounds and to determine relative abundance by comparing diffraction data to a database maintained by the International Centre for Diffraction Data. X-ray photoelectron spectroscopy (XPS) (ESCALAB 250, Thermo Scientific) was applied to characterize the adsorbed species on the surface of the above samples. The water contact angles of SR and three kinds of C-SRs were measured with a drop shape analysis system (DSA100, Krüss) in the sessile mode at room temperature. The results of surface characteristic are shown in Figure 1.

### Cell culture

The human dermal fibroblasts (Cell Bank of Chinese Academy of Sciences, Shanghai, China) were maintained in DMEM medium (Sigma, St.Louis, MO, USA), supplemented with a 10% fetal bovine serum (FBS, Invitrogen, Carlsbad, CA, USA), penicillin/Streptomycin (1∶100, Sigma, St.Louis, MO, USA) and 4 mM L-glutamine (Sigma, St.Louis, MO, USA), in a CO_2_ incubator at 37°C.

### Cell adhesion and Cell Counting Kit-8(CCK-8) cell viability assay

Human dermal fibroblasts were collected and seeded into 24-well multi-plates with or without SR and C-SRs (sample diameter 14 mm), at a density of 5×10^4^ cells/well and cultured for 6 h. The culture medium was removed and cells were washed twice with PBS. Then adherent cells were collected by trypsinization and diluted with a trypan blue/DMEM mixture after centrifugation (1000 rpm, 200 g, 5 min) and counted with a hemacytometer. Cell adhesion rate was calculated from the proportion of adherent/seeded cells.

Human dermal fibroblasts (1×10^6^) were collected and seeded at a density of 1×10^3^ cells/well in 96-well plate pre-coated with SR or indicated C-SRs. The cells were further cultured in growth medium in 37°C incubator for 24, 48 and 96 hours. Cell viability was then measured using a CCK-8 (Dojindo, Japan) according to manufacturer's protocol [20]. The OD value of cells cultured on C-SR surface was normalized to that of cells cultured on SR surface. All experiments repeated six times with 6 well for each condition.

### Cell morphology and adhesion proteins observation by SEM and immuno-fluorescence

As described previously [9], human dermal fibroblasts were cultured on SR or C-SR surface as monolayer, and then rinsed with PBS and fixed with 3% buffered glutaraldehyde for 20 min at 4°C. Then samples were dehydrated with aqueous ethanol (30–100%) step by step. Samples were lyophilized and coated with platinum. Cell morphology was observed by SEM (AMRAY 1000-B, Amray Inc, Bedford, Mass, USA).

**Figure 2:**
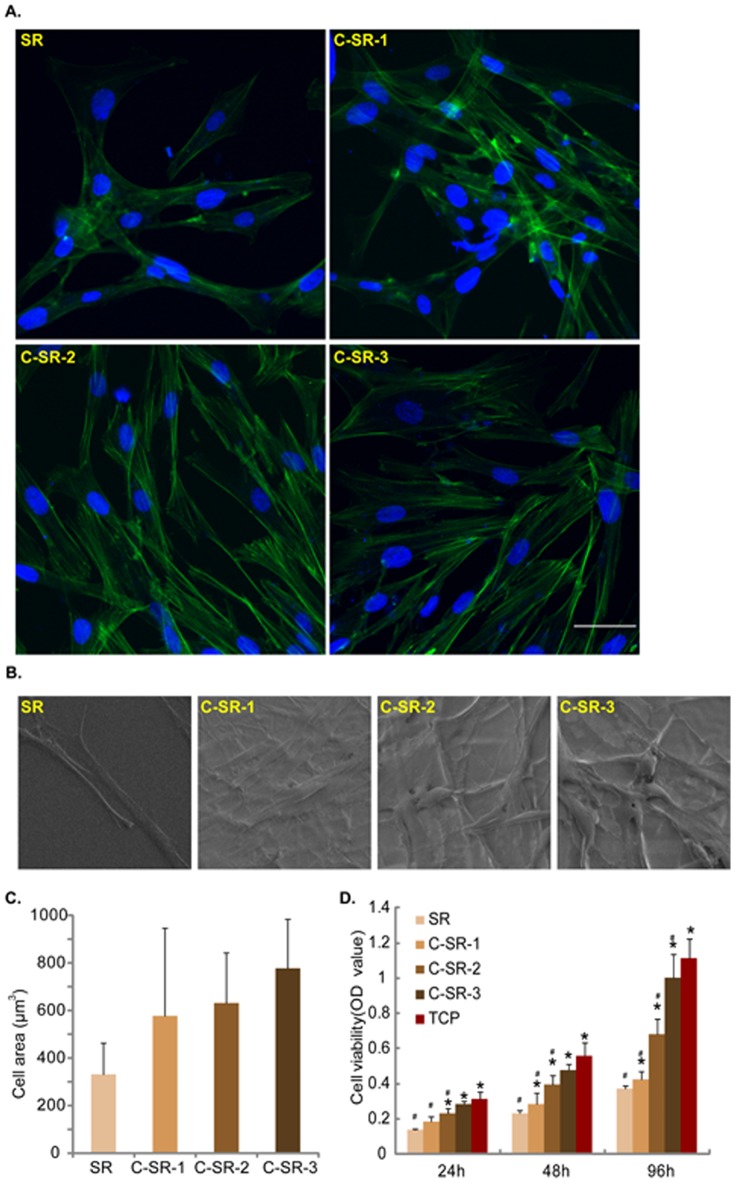
Human dermal fibroblasts cultured on C-SR grow faster and showed a more fibroblastic appearance. Dermal fibroblasts (1×10^6^) were seeded in 6-well plate pre-coated with SR or three C-SRs (C-SR-1, C-SR-2 and C-SR-3). The cells were incubated in 37°C incubator for 48 hours for observation of cell morphology. (A) FITC-labeled actin tracer was used to observe cytoskeleton by immuno-fluorescence microscopy. Fibroblasts cultured on C-SRs had more and larger filopodia spreading out, and their microfilament stretched longer and arranged more regularly. (B) SEM images further demonstrated a more fibroblastic appearance of fibroblasts cultured on C-SRs. (C) Cell area depicted higher values for cells on C-SRs than those on SR. But the difference was not statistically significant between any two groups (p>0.05). (D) Dermal fibroblasts were cultured in 96-well plate (at a density 1×10^3^ cells/well) for 24, 48 and 96 hours. The cell viability was analyzed by CCK-8 assay. The cell viability was also increased by C ion implantation. *p<0.05 The difference was statistically significant when the substrate was compared to SR at any time point. ^#^p<0.05 The difference was statistically significant when the substrate was compared to TCP at any time point. Experiments in this figure were repeated three times, similar results were obtained each time. Bar = 20 µm. All data are expressed as mean ± SD (error bars).

**Figure 3:**
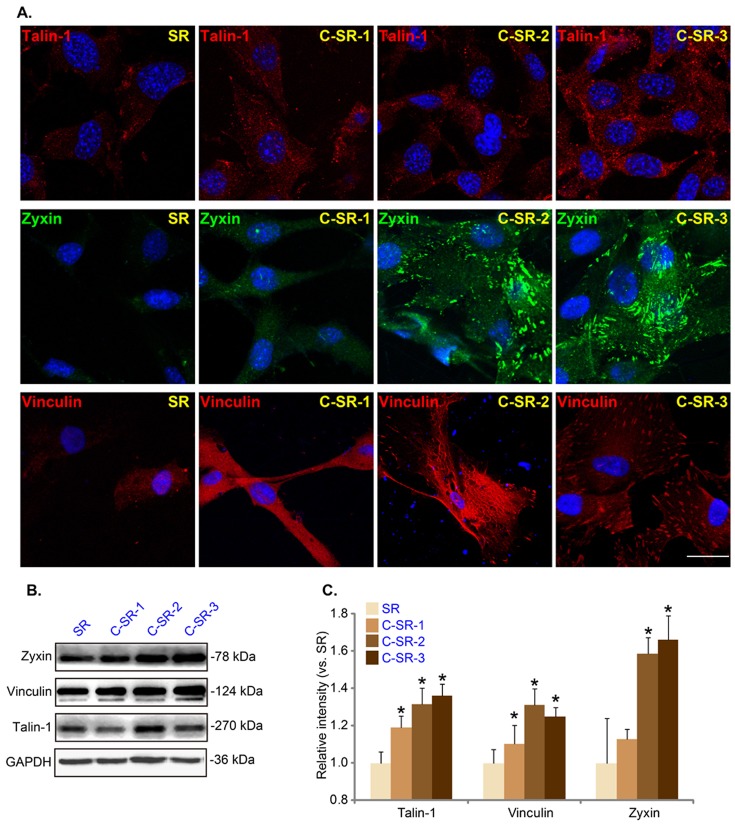
Dermal fibroblasts adhere more strongly to the surface of C-SR with increased expression levels of adhesion proteins. Immuno-fluorescence results (A) and western blots results (B and C) show the expressions and localization of talin-1, zyxin and vinculin in dermal fibroblasts cultured on SR or different C-SRs coated 6-well plate (48 hours). Note that there were significantly more expression levels of talin-1, zyxin and vinculin in dermal fibroblasts cultured on C-SRs than in other cells. *p<0.05 The difference was statistically significant when the substrate was compared to SR for detecting different adhesion protein. These experiments were repeated three times, similar results were obtained each time. Bar = 20 µm.

**Figure 4:**
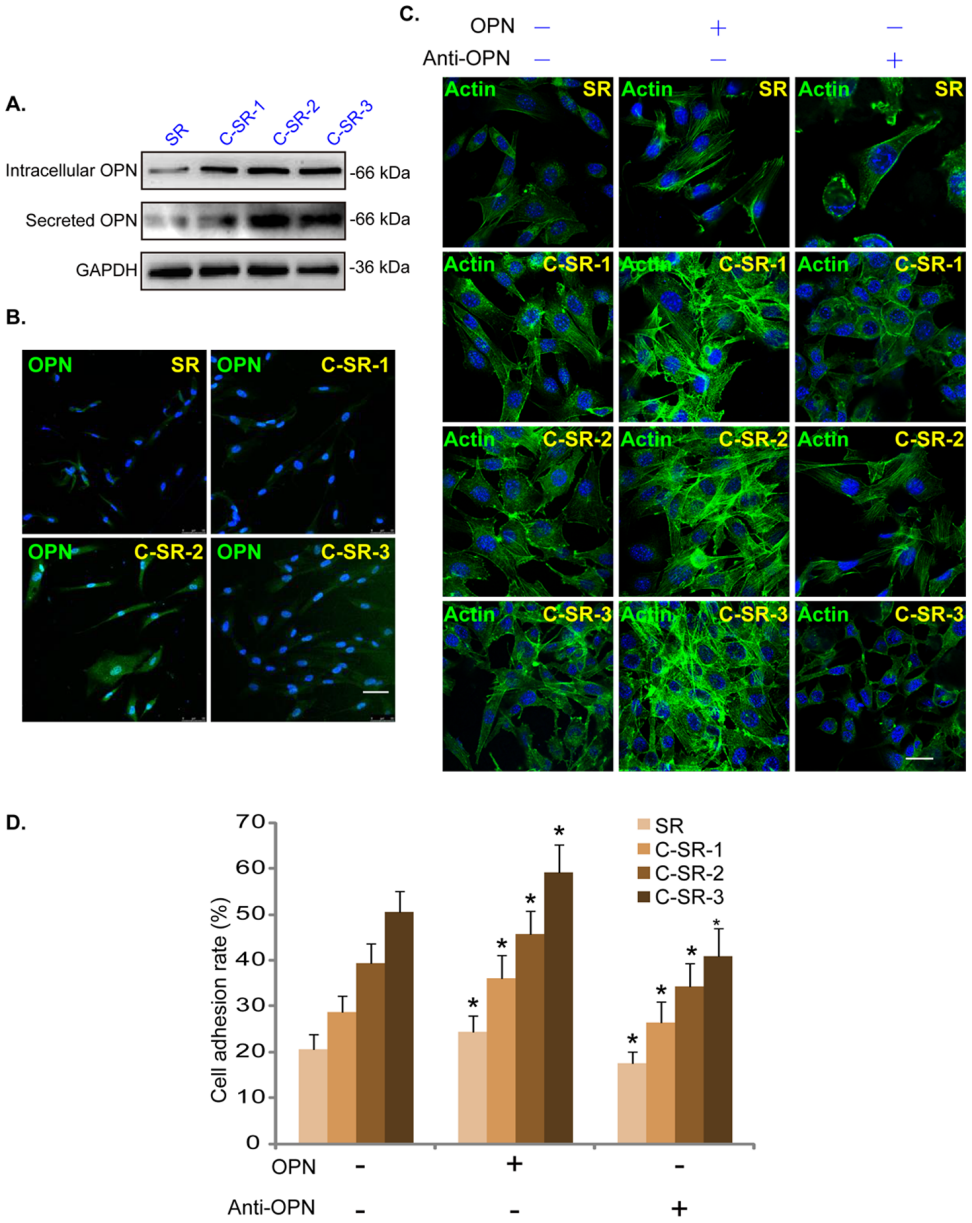
OPN acts as an important protein to promote cell adhesion on the surface of C-SR. (A) Western blots results show the expression level of OPN secreted from dermal fibroblasts cultured on SR or C-SR coated 6-well plate (48 hours). Note that OPN was up-regulated and secreted from dermal fibroblasts cultured on C-SRs. (B) Immuno-fluorescence images show that the expression level of OPN in dermal fibroblasts cultured on C-SR coated 6-well plate was increased (48 hours). (C) FITC-labeled actin tracer results demonstrate that exogenously-added purified OPN (0.5 µg/ml) enhanced dermal fibroblasts adhesion and proliferation. On the other hand, the monoclonal antibody against OPN inhibited dermal fibroblasts adhesion and proliferation on the surface of C-SRs (48 hours). (D) Cell adhesion on different substrates. OPN promoted fibroblasts adhesion, and monoclonal antibody against OPN significantly inhibited cell adhesion. However, the difference was not statistically significant when the substrate in group (OPN− Anti-OPN−) was respectively compared to the substrate in group (OPN+ Anti-OPN−) or group (OPN− Anti-OPN+) (p>0.05). The difference was significant between the substrates in group (OPN+ Anti-OPN−) and group (OPN− Anti-OPN+) (*p<0.05). These experiments were repeated three times, similar results were obtained each time (Fig. 4A, Fig. 4B and Fig. 4C). Bar = 20 µm. The cell adhesion test was performed six times, the data are expressed as mean ± SD (Fig. 4D).

**Figure 5:**
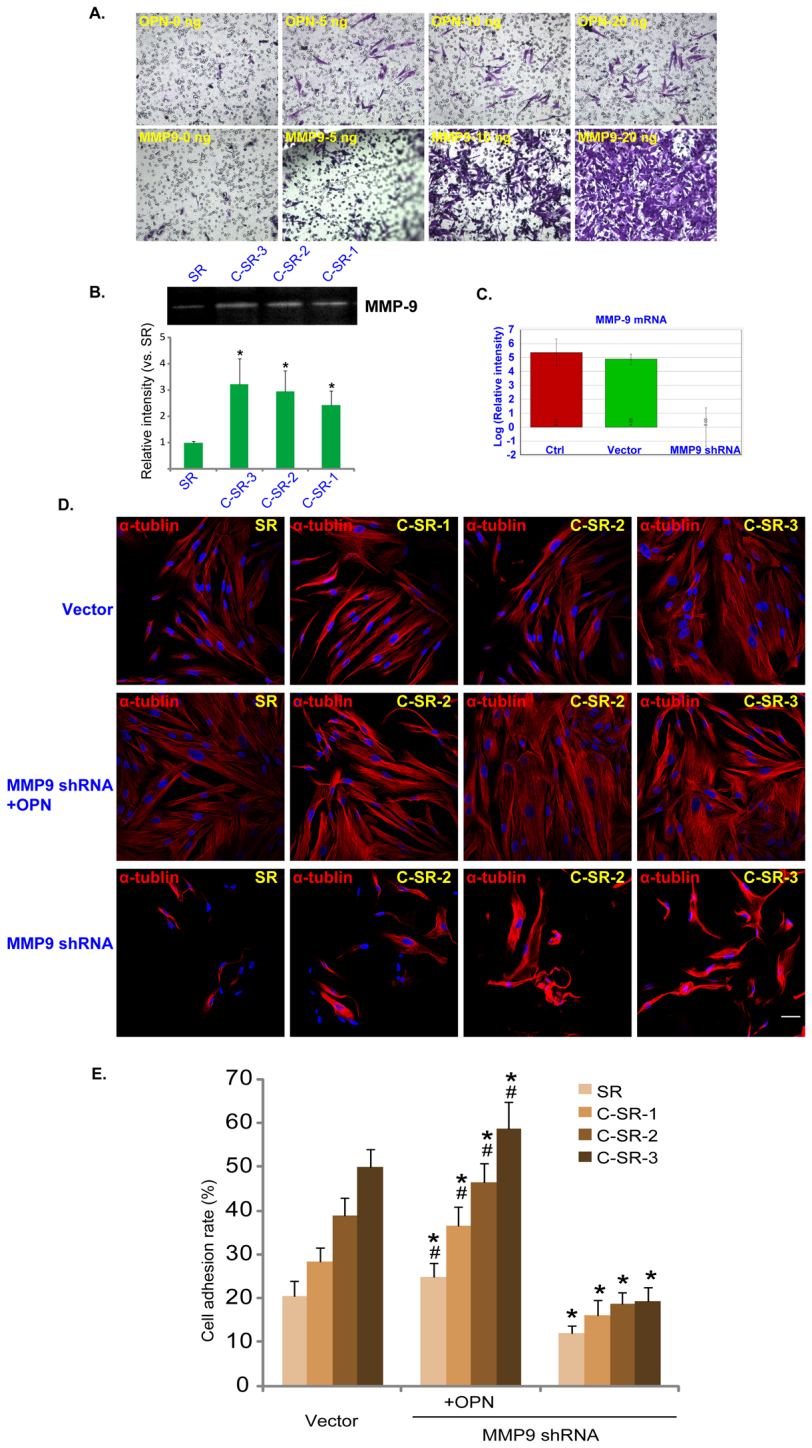
MMP-9 is important for dermal fibroblast adhesion and migration on the surface of C-SR. (A) Transwell results show that exogenously-added OPN and MMP-9 facilitated dermal fibroblasts migration (48 hours). (B) Zymography assay shows the MMPs activity of fibroblasts cultured on SR or different C-SR coated 6-well plate (48 hours). The difference was statistically significant when any C-SR was compared to SR (*p<0.05). (C) MMP-9 mRNA level in control dermal fibroblasts or in fibroblasts infected with MMP-9-shRNA or scramble-shRNA containing lentiviral particles. (D and E) Immuno-fluorescence results show that MMP-9 shRNA inhibited fibroblast adhesion and growth on the surface of C-SR and it was rescued by exogenously-added purified OPN (20 ng). *p<0.05 The difference was statistically significant when the substrate in group (MMP9 shRNA or MMP9 shRNA+OPN) was compared to the substrate in group (vector). ^#^p<0.05 The difference was also statistically significant when the substrate in group (MMP9shRNA+OPN) was compared to the substrate in group (MMP9shRNA). These experiments were repeated three times, similar results were obtained each time. Bar = 20 µm. The cell adhesion test was performed six times, the data are expressed as mean ± SD (Fig. 5E).

As described previously [21], [22], human dermal fibroblasts were cultured on SR or C-SR surface as monolayer on cover slips and were fixed in cold paraformaldehyde (4%) for 15 min at −4°C, washed three times with PBS. Afterwards, cells were blocked with 5% BSA in PBS (pH 7.5) for 30 min, followed by overnight incubation with the primary antibody. The corresponding secondary antibody was then added for 1 h at room temperature. In Figure 2, the cytoskeleton was stained with FITC-labeled actin Tracker probes (Beyotime, Shanghai, China). In Figure 3A, the primary antibodies were rabbit anti-talin-1 (1∶500, Abcam, Cambridge, England), rabbit anti-zyxin (1∶500, cell signaling, Danvers, MA, USA) or mouse anti-vinculin (1∶500, Sigma-Aldrich, St.Louis, MO, USA) and the corresponding Cy3-tagged secondary antibodies to talin-1 and vinculin (Beyotime, Shanghai, China) and FITC-tagged secondary antibody (Invitrogen, Shanghai, China) to zyxin were used. In Figure 4B, rabbit anti-OPN (1∶100, Santa Cruz Biotech, Santa Cruz, CA, USA) was used as primary antibody and FITC-tagged secondary antibody (Invitrogen, Shanghai, China) was used. In Figure 4C, the cytoskeleton was also stained with FITC-labeled actin Tracker probes (Beyotime, Shanghai, China). In Figure 5D, rabbit anti-α-tublin (1∶1000, millipore, MA, USA) was used as primary antibody and the corresponding Cy3-tagged secondary antibody (Beyotime, Shanghai, China) was used to detect cytoskeleton. The cell nuclei were stained with 4′, 6′-diamidino-2-phenylindole (DAPI, 0.5 µg/ml) (Sigma-Aldrich, St.Louis, MO, USA) in Figure 2, 3, 4 and 5. Cells were visualized through a Leica confocal microscope with the appropriate filters. All the experiments were repeated six times and 6 slides for each condition. Cell surface area for comparing cell shape was measured by Image-Pro plus 6, six cells of each sample were selected randomly for quantitative comparison.

### Protein isolation, western blot and data quantification

As described before [22]–[24], dermal fibroblasts were seeded on the 6-well plate coated with SR or C-SRs for 48 hours. Afterwards, cells were washed with ice-cold PBS and lysed using lysis buffer (pH, 7.4) containing 1% Nonidet P-40 (NP-40), 1% deoxycholate, 0.1% sodium dodecyl sulfate, 150 mmol/L NaCl and 10 mmol/L Tris-HCl. The lysates were collected and centrifuged. The concentration of the extracted protein was measured by bicinchoninic acid assay kit (catalogue B9643, Sigma-Aldrich, St.Louis, MO, USA). Aliquots of 30–40 µg of protein from each sample (treated as indicated in the legends) were separated by 10% SDS–polyacrylamide gel electrophoresis (SDS-PAGE) and transferred to the polyvinylidene difluoride (PVDF) membrane (Millipore, MA, USA). After blocking with 10% instant nonfat dry milk for 1 hour, the membrane was incubated with specific antibody overnight at 4°C, followed by incubation with secondary antibodies (HRP-conjugated) for 60 min at room temperature. The antibody binding was detected with the enhanced chemiluminescence (ECL) detection system (Amersham Biosciences, Piscataway, NJ, USA). The primary antibodies used in this study were as follows: rabbit anti-OPN (1∶300, Santa Cruz Biotech, Santa Cruz, CA, USA), rabbit anti-zyxin (1∶1000, cell signaling, Danvers, MA, USA), rabbit anti-talin-1 (1∶1000, Abcam, Cambridge, England), mouse anti-vinculin (1∶5000, Sigma-Aldrich, St.Louis, MO, USA) antibodies, and the anti-GAPDH (the house keeping gene, 1∶1000, Sigma-Aldrich, St.Louis, MO, USA). The OPN in the supernatant of dermal fibroblast culture medium was also tested by western blot. The intensity of each blot was quantified by Image J software, and was normalized to the loading control (GAPDH). Each experiment was repeated at least three times, with 3 wells per replicate.

### Zymography

The MMP-9 activity was analyzed using SDS-PAGE substrate gels. Gelatin (Bloom 300, Sigma-Aldrich, St.Louis, MO, USA) was added to 10% acrylamide separating gel at a final concentration of 1 mg/ml. Samples containing equal amount proteins were mixed with non-reducing sample buffer (62.5 mM Tris-HCl, pH 6.8, 10% glycerol, 2% SDS, 0.1% bromophenol blue), and were added to the gel wells without denature (boiling). Following electrophoresis, gels were washed twice in 2.5% Triton X-100 for 30 min at 37°C to remove the SDS. Gels were further incubated at 37°C overnight in the developing buffer containing 50 mmol/L Tris-HCl, 0.2 mol/L NaCl, 5 mmo/L CaCl_2_ and 0.02% Triton X-100. Gels were stained with 0.5% coomassie blue in 30% methanol, 10% glacial acetic acid for 30 min, and were then de-stained in the same solution but coomassie blue. Gelatin-degrading enzymes were identified as clear bands against the blue background of the stained gel. Images of stained gels were captured under illumination using the BIO CHEN Imagestroe 7500. The intensity of the bands was measured by densitometric analysis, experiments were repeated three times at least. MMP-9 activity level in C-SR group was expressed in the form of fold changes vs. SR group.

### Total RNA isolation and real-time reverse transcriptase polymerase chain reaction (RT-PCR)

The expression of MMP-9 mRNA was analyzed by real-time reverse transcription-polymerase chain reaction (RT-PCR). Total RNA was prepared from cultured cells using TRIzol reagent (Invitrogen, CA, USA) according to the manufacturer's instructions. The concentration and purity of RNA was measured spectrophotometrically at A260 and A280. RT-PCR was performed using TOYOBO ReverTra Ace RT-PCR kit according to the manufacturer's instruction. The resulting cDNA was used as a template for PCR with specific primer pairs using Primer Premier 5.0 software (Premier Biosoft, International, Palo Alto, CA, USA). The results were analyzed using delta Ct. All real-time PCRs were performed three times at least.

### Generation of MMP-9 knockdown stable dermal fibroblasts by lentiviral infection

The dermal fibroblasts were seeded in a 6-well plate with 60% confluence in growth medium with polybrene. Twenty µl/ml of lentiviral particles containing MMP-9 shRNA (Santa Cruz Biotech, Santa Cruz, CA, USA) were added to the cells for 24 h, cell culture medium was then replaced by growth medium and cells were cultured for another 24 h, stable clones expressing MMP-9 shRNA were selected using puromycin (1 µg/ml). The culture medium was replaced with fresh puromycin-containing culture medium every 2–3 days, until resistant colonies could be identified. The MMP-9 mRNA expression in the transfected cells was detected by RT-PCR in the resistant colonies. Same amount of scramble non-sense shRNA lentiviral particles (Santa Cruz Biotech, Santa Cruz, CA, USA) was added to the control cells.

### Transwell assay

Fibroblasts were maintained for 24 h in serum-free medium, prior to treatment with OPN (0 ng/L, 5 ng/L, 10 ng/L, 20 ng/L) (Proteintech Group, Chicago, IL, USA) or MMP9 (0 ng/L, 5 ng/L, 10 ng/L, 20 ng/L) (Proteintech Group, Chicago, IL, USA) for a further 24 h. Fibroblasts (100 µl in DMEM, 1×10^5^ cells/ml) were then plated onto upper chamber in a 24-well plate, according to the manufacturer's instructions (Millipore, MA, USA). After migration for 24 h, penetrated cells on the filters were fixed in dried methanol, stained in 0.1% crystal violet, washed by PBS, and then photographed in each group.

### Statistical analysis

The data presented in this study was expressed as means ± SD (standard deviation). Statistical difference was analyzed by one-way *ANOVA* followed by multiple comparisons performed with post hoc Bonferroni test (SPSS version 16.0). Value of *p*<0.05 was considered statistically significant.

## Results

### C ion implantation changes the physical and chemical properties of SR surface

C-SR-1, C-SR-2 and C-SR-3 were generated as described. SEM and AFM were applied to observe the surface micro-morphology of SR and three different C-SRs. The SEM results failed to find any significant differences between SR and three C-SRs (data not shown), indicating that C ion implantation didn't change the macro-scale surface of SR. However, results from the AFM images revealed that the surfaces of C-SRs were composed of larger irregular peaks and deeper valleys, while SR exhibited a relatively smooth and more homogeneous surface (Figure 1A). The surface roughness of C-SR-3, which underwent most C-ion implantation, was highest among all three C-SRs (Figure 1A). Thus, C ion implantation on the SR changed the surface microstructure. To further estimate the physical and chemical properties C-SRs, XPS analysis was employed. Results showed that C ion implantation significantly changed the surface charge distribution, silicone oxygen rate and chemical-element distribution of SR, all of which could facilitate cell adhesion (Figure 1B and C). Note that with the ion implantation dose increasing, the C content in the material increased, while the Si content decreased (Figure 1C), suggesting that implanted C atom may replace the Si of SR, interrupting the original Si-O assemble, so the surface free energy increases, thereby theoretically decreasing material's water contact angle. The XRD images exhibited characteristic peaks (black arrow) pattern with crystalline materials in both SR and C-SRs. The distinct XRD patterns among SR and C-SRs, or among three C-SRs may be attributable to different amount of C ion implantation. It is notable that the difference was very small (Figure 1D). FTIR analysis was performed to clarify the formation of new bonding motifs. The spectrums for C-SRs are similar to that for pristine SR (Figure 1E). These results of XPS, XRD and FTIR indicate that the C ion implantation may interrupt the silicon bonds, but the interaction between C ion and SR is weak. To test the hydrophilicity/hydrophobicity of these materials, water contact angle was also examined. We found that C ion implantation significantly decreased the water contact angle of SR (Figure 1F), and C-SR-3 had lowest water contact angle among all C-SRs (Figure 1F). These results together indicate C ion implantation significantly changes the physical and chemical properties of SR surface, to possibly favor an improved biocompatibility.

### Human dermal fibroblasts cultured on C-SR grew faster and had a more fibroblastic appearance

From the cytoskeleton images in Figure 2A, we found that human dermal fibroblasts cultured on the C-SR grew faster, and showed a more fibroblastic appearance. These cells had more and larger filopodia spreading out, and their microfilament stretched longer and arranged more regularly (Figure 2A). While cells cultured on the regular SR grew slower, and had a “narrow” shape (Figure 2A). SEM image results further demonstrated a more fibroblastic appearance of fibroblasts cultured on C-SRs (Figure 2B). We also quantified the morphology of fibroblasts on different substrates. The cell area depicted higher values for cells on C-SRs than those on SR, and it was positively correlated with implanted C ion doses. But there was no statistically significant difference (p>0.05) (Figure 2C). Results in Figure 2D showed that dermal fibroblasts cultured on the C-SRs grew faster, and had higher cell viability. Cells cultured on C-SR-3 had the highest viability among SR and all C-SRs, but lower than tissue culture plates (TCP) (Figure 2D). These results together indicate that C ion implantation could provide a better environment for cell adhesion and growth.

### Dermal fibroblasts adhere more strongly to the surface of C-SR with increased expressions of many adhesion proteins

Cell adhesion process is associated with increased expressions of many adhesion-associated proteins, including talin-1, zyxin and vinculin. These proteins are involved in the formation of focal adhesion complexes, acting as a conjugation site for both cytoskeleton organization and integrin signaling transduction, which is a critical step for cell adhesion/migration initiation. Immuno-fluorescence results revealed that dermal fibroblasts cultured on C-SRs expressed higher levels of talin-1, zyxin and vinculin than cells grew on SR (Figure 3A). In addition, western-blot and relative intensity analysis showed that with the implanted C ion dosage increasing, the expression levels of above proteins were higher (Figure 3B and 3C). These results and results in Figure 2 suggested that the growth of dermal fibroblasts on C-SRs is better than those on SR, and the expression levels of many adhesion proteins are increased.

### OPN works as an important protein to promote cell adhesion on the surface of C-SR

OPN originally isolated from bone, is a multifunctional acidic glycoprotein. It is secreted from cells, works as both an ECM component and a soluble cytokine [25], and is able to bind to both RGD-containing integrins (αvβ1, αvβ3, αvβ5, αvβ1, α8β1) or non-RGD integrins (α4β1, α9β1) to promote cell survival, proliferation, migration, invasion, and metastasis [26]. Western blot results in Figure 4A showed that dermal fibroblasts cultured on C-SRs had higher OPN expression and secretion than those cultured on SR. Immuno-fluorescence images in Figure 4B further confirmed higher intracellular OPN expression in those dermal fibroblasts. Importantly, exogenously-added OPN promoted dermal fibroblast adhesion on both SR and C-SR surfaces. On the other hand, the monoclonal antibody against OPN significantly inhibited cell adhesion on the C-SR surfaces (Figure 4C). However, the difference was not statistically significant when the substrate in group (OPN− Anti-OPN−) was respectively compared to the substrate in group (OPN+ Anti-OPN−) or group (OPN− Anti-OPN+) (p>0.05). The difference was significant between the substrate in group (OPN+ Anti-OPN−) and group (OPN− Anti-OPN+) (* p<0.05). These results together suggest that dermal fibroblasts cultured on C-SR surface express and secrete higher level of OPN, which helps cell adhesion.

### MMP-9 is important for dermal fibroblast adhesion and migration on the surface of C-SR

MMP can promote ECM degradation during physiological and pathological tissue remodeling processes. Meanwhile, MMP-9 is also important for cell migration and skin wound healing. Transwell experiment demonstrated that both exogenously added human recombination MMP-9 and OPN promoted dermal fibroblasts migration (Figure 5A). Significantly, dermal fibroblasts cultured on the C-SR surfaces showed higher activity of MMP-9 by zymography (Figure 5B). After a stable MMP-9 knockdown dermal fibroblast line was created (Figure 5C), cell adhesion was largely inhibited on both SR and C-SR surfaces, which was rescued by OPN (Figure 5D and Figure 5E). These results together indicate that MMP-9 is important for dermal fibroblast adhesion and migration on the surface of C-SR.

## Discussions

There are many ways to remodel the surface of SR, including microwave plasma surface modification [27], copolymerization [28] and others. By using indirect copolymerization, studies have integrated multiple drugs or related compounds onto the SR surface to improve its biocompatibility. For example, halofuginone, an anti-fibrotic drug was attached on the surface of silicone breast implants to reduce the capsular fibrosis [29]. Oxygen/ammonia plasma and poly (ethylene-alt-maleic anhydride) (PEMA) were also added to SR to improve surface functionality to permanently improve adhesion [30]. Meanwhile, scientists have used the chemical vapor deposition method to attach some biocompatible compound to the surface of SR. Poly (o-amino-p-xylylene-co-p-xylylene) (amino-PPX) and polyacrylamide (PAAm) were introduced onto SR to help improve its surface functions [2]. For all the methods mentioned above, the manufacture procedures are complicated and contain multiple steps, conditions are relatively harsh, which makes the industrial mass manufacture almost impossible.

Ion implantation is often used in semiconductor device fabrication and metal finishing, as well as various applications in materials science research [31]–[35]. Ion implantation affects the morphology and structure of the material, and it is dependent on the source of energy, energy flow, the beam and the composition of the target material [32], [33]. When the ions are implanted into the substrate materials, the ions will collide with the target atoms. The collision processes may have three different outcomes: nuclear collision, electron collision, and charge exchange. Incident ions lose energy during every collision process and could be stopped within the materials, where they act as impurities. Most of these incident ions stay at the interstitial sites, and these interstitial impurities may migrate to substitution positions after annealing. This substitution doping gives the substrate materials better properties [34]. Sputtering effect is another important phenomenon. This effect generally impacts the shape and morphology of substrate materials. During the implantation process, the incident ions induce collision cascades, the atoms of the target material may get enough energy to be ejected out from the substrate material [35]. On this account, the surface region of the smooth substrate will be sputtered away. This sputtering effect will be enhanced at low-lying areas, and then the substrate will become rougher.

In the current study, we successfully modified SR surface through C ion implantation. It is well-known that surface characteristics play a vital role in the functions of biomaterials [36], [37]. That is to say, the biological response to biomaterials is largely controlled by their surface characteristics, including chemistry, morphology, hydrophilicity/hydrophobicity, *etc*. So it is important to understand how C ion implantation impacts the surface characteristics of SR. C ion implantation increased the roughness of SR (Figure 1A), changed surface chemistry (Figure 1B and Figure 1C) and decreased water contact angle (Figure 1F). The AFM micrographs results showed that the surfaces of C-SRs were composed of large, irregular peaks and deep valleys, and these newly formed C-SRs had decreased water contact angle. Significantly, with more C ion implanted, the C-SR surface roughness increased and the hydrophobicity decreased. On rough surfaces, good cell adhesion/attachment and proliferation can be achieved [38], but decreased cell functions have also been reported [39]. Other factors such as hydrophilicity/hydrophobicity and surface chemistry are also considered important [40]. Compared with hydrophobic surface, hydrophilic surface is feasible for cell adhesion/attachment [40]. In our study, the dermal fibroblasts cultured on the surface of C-SRs grew faster, and were with higher mobility and better viability than those cultured under other conditions. Meanwhile, the cytoskeleton alignment was improved. All these results indicated that the cell adhesion/attachment on this modified surface was significantly improved. These results reveal that C ion implantation changes the surface roughness and water contact angle, thus the biocompatibility is improved, in accordance with previous studies [38].

Besides surface morphology and hydrophilicity/hydrophobicity, surface chemistry is also considered quite important on cell behavior [37], [41]. Moreover, small amounts of foreign atoms or molecules on the surface can dramatically alter the surface reactivity [42]. As ECM is crucial to mediating cell adhesion/attachment onto the surface of implants [43]. And the cellular response to material involves a chain of complex biological reactions including protein adsorption, receptor-ligand binding and signal transduction. So the improvement of cyto-compatiblity is possible due to preliminary ECM protein adsorption on C-SRs, and subsequent signal transduction [44]. In the current study, we found a significant increased intracellular expression level of OPN, as the intracellular response. Meanwhile, we also found increased OPN in culture media, which was secreted by dermal fibroblasts cultured on the surface of C-SRs and will be absorbed on the surface of C-SRs. The pleiotropic nature of OPN allows it to promote cell adhesion, migration, survival and others, which may be due to its ability to interact with a variety of cell types via binding multiple integrins and activating many signaling pathways [45], [46]. Furthermore, we found that the improved cells adhesion, migration and proliferation seen in those cells were largely inhibited by the monoclonal antibody against OPN. In a previous study, the increased expression level of OPN was observed in the early stage after skin wound, and it also displayed a possible fibrosis gene spatial profile with expression in deep dermal cell layers [47]. Based on our results and previous studies, we speculate that OPN is an important candidate among ECM proteins, will be absorbed onto the surface of C-SR when it is implanted subcutaneously, in accordance with future clinical application, and will play an important role in cell adhesion, growth and migration.

As a non-collagenic ECM protein, OPN exists both as a component of the ECM and as a soluble cytokine [45]–[48]. OPN contains an arginine-glycine-aspartate (RGD) domain, which promotes cell adhesion by binding integrins, and leads succedent intracellular signal transduction to promote cell proliferation and migration, *etc* [45], [46], [49]. However, the molecular mechanism behind OPN regulation of cell migration is not well understood. Several studies have reported that OPN may promote MMPs secretion to enhance cell migration. Recent studies have confirmed that OPN increases cell adhesion and migration through inducing MMP-9 or MMP-2 up-regulation and secretion in different cells [50], [51]. In the current study, a high MMP-9 activity was observed in fibroblasts grew on the surface of C-SRs. Meanwhile, both recombinant OPN and MMP-9 promoted cell migration. Importantly, the high mobility and adhesion abilities seen in cells were inhibited by MMP-9 RNAi, and such an effect was reversed by exogenously-added OPN. These results suggeste that MMP-9 is the downstream signal molecular of OPN, playing an important role in the migration of fibroblasts which are seeded on C-SRs.

## Conclusions

Carbon (C) ion implantation was here used to modify silicone rubber (SR). The results of the present study suggest that C ion implantation significantly improves the cyto-compatibility of SR. The enhancement is attributable to the surface characteristics, including surface roughness, surface chemistry, and hydrophilicity/hydrophobicity. OPN was found to act as an important protein to promote cell adhesion to the surface of C-SR.
